# Alternative Reproductive Tactics in the Shell-Brooding Lake Tanganyika Cichlid *Neolamprologus brevis*

**DOI:** 10.1155/2012/193235

**Published:** 2012-07-19

**Authors:** Kazutaka Ota, Mitsuto Aibara, Masaya Morita, Satoshi Awata, Michio Hori, Masanori Kohda

**Affiliations:** ^1^Department of Zoology, Kyoto University, Kitashirakawa-Oiwake, Sakyo, Kyoto 606-8502, Japan; ^2^Department of Biology and Geosciences, Osaka City University, Sugimoto, 3-3-138 Sumiyoshi, Osaka 558-8585, Japan; ^3^Graduate School of Bioscience and Biotechnology, Tokyo Institute of Technology, 4259 Nagatsuta, Yokohama 226-8501, Japan; ^4^Graduate School of Fisheries Sciences, Hokkaido University, 3-1-1 Minato, Hakodate, Hokkaido 041-8611, Japan; ^5^TBRC Sesoko Station, University of the Ryukyus, 3422 Sesoko, Motobu, Okinawa 905-0227, Japan; ^6^Sado Marine Biological Station, Niigata University, 87 Tassha, Sado, Niigata 952-2135, Japan

## Abstract

Alternative reproductive tactics (ARTs) are found in several Lake Tanganyika shell-brooding cichlids. Field studies were conducted in the Wonzye population to examine reproductive ecology and ARTs in the Lake Tanganyika shell-brooding cichlid *Neolamprologus brevis*. We discovered that this fish occurred in both rocky- and sandy-bottom habitats, but in rocky habitats, brood-caring females exclusively occurred in shell-patches that another cichlid species created. All *N. brevis* of both sexes in the patches were sexually mature, whereas immature males and females with unripe eggs were found frequently in sandy-bottom habitats. Males in sandy-bottom habitats were smaller, but fed more frequently and were in better somatic condition than males in the patches. Similar tendency was found in females. This indicates that *N. brevis* uses different habitats depending on the stage of its life history, with migration from sandy-bottom habitats to the shell-patches for reproduction. Males in the patches exhibited different behavior patterns: floating above the patches and lying in the patches. The former was larger, more aggressive, and invested less in gonads (relative to body size) than the latter. These results accord with those of other shell-brooding Lake Tanganyika cichlids with ARTs, and they therefore suggest the presence of ARTs in *N. brevis*.

## 1. Introduction

In species where males hold the resources required for breeding, reproductive behavior is often associated with “bourgeois” territorial tactics that involve defense of resources in an attempt to monopolize mating opportunities [1–4]. Bourgeois tactics are usually adopted by males who achieve competitive superiority (and also attractiveness) thorough behavioral (e.g., antagonistic behaviors) and morphological investments (e.g., large body size). These exclude less competitive males from reproduction. Such intrasexual competition for mating may lead to the evolution of alternative reproductive tactics (ARTs) [5, 6]. Competitively inferior, less attractive, and smaller subordinate males are unlikely to monopolize opportunities for mating, but they may evade monopolization by bourgeois males by using reproductively “parasitic” tactics that often involve “sneaky” behavior and the theft of reproductive efforts by bourgeois males [1, 3, 4, 6].

ARTs inherently give rise to sperm competition, that is, a competition among sperm from different males for the fertilization of ova [7]. Subordinate males exhibiting “sneaky” mating behavior (i.e., sneakers) face a high probability that their sperm will encounter sperm competition (sperm competition risk), as they obtain opportunities for fertilization by taking part in the mating of others. However, this is not necessarily true for bourgeois males, which engage in mating without any rivals (low sperm competition risk) unless sneakers frequently intrude in their mating. The theoretical game model of sperm competition predicts that sneakers are forced to make a greater investment in ejaculate than are bourgeois males due to the increased risk of sperm competition [8]. Differential testes investment, as well as behavioral and morphological investments, are found in many species with ARTs (e.g., [9–15]; see also [1, 16–18], for review). 

ARTs have been reported in a variety of taxa [4, 6] and particularly in fish [1, 3, 16]. ARTs are prevalent and diversified in the Lake Tanganyika cichlid tribe Lamprologini [19], although such ARTs can also be found in other Lake Tanganyika tribes ([20] and references therein). In the Lamprologini, ARTs are typically dichotomous, that is, bourgeois territorial and parasitic “sneaky” tactics (e.g., cooperative brooders, *Julidochromis ornatus* [21], *Julidochromis transcriptus* [22, 23], and *Neolamprologus pulcher* [24]; a shell brooder, *Lamprologus callipterus* [3, 25–27]; a rock-hole brooder, *Telmatochromis temporalis *[28]). In *T*. *temporalis*, piracy tactic (i.e., the takeover of spawning events from territorial males by the largest males) also appears but seasonally [29]. In the shell-brooder* Telmatochromis vittatus*, four reproductive tactics have been reported (sneaker, satellite, territorial, and piracy tactics, [30]), with piracy tactics being dependent on conditions [31]. Furthermore, mixed paternity is found in biparental breeders (*Neolamprologus meeli* [32] and *Variabilichromis moorii *[33]), suggesting the presence of parasitic tactics.

A Lamprologini cichlid,* Neolamprologus brevis* (a synonym of *Neolamprologus calliurus* [34]), is characterized as an obligate shell brooder that spawns and cares for broods inside empty gastropod shells [25]. The shell is also used as shelter when these fish encounter predators [35]. The Wonzye population, which is located in a southern region of the lake, occurs in a wide range of habitats in the littoral zone, from shallow rocky habitats to relatively deep offshore sandy-bottom habitats. However, ecological information for this fish is only available from a particular habitat, shell patches consisting of a number of gastropod shells (mean = 96 shells, [25]). It is not known how this fish uses other habitats. The patches are not spontaneous but are created by nesting males of* L*.* callipterus* [36]. *L*.* callipterus* is the only species that can transport shells. A female *L*. *callipterus* occupies a shell for breeding, which lasts for 10–14 days, during which period she spawns and subsequently provides brood care [36]. *L*.* callipterus* females that are ready to spawn or care for broods are not found in the patches [25], suggesting that they leave the patches immediately after the completion of a breeding event. *N*.* brevis* also breeds in the patches, and a number of females and several males are found at the same time, suggesting that this fish has a multimale polygynous mating system [25]. Two other species, *Neolamprologus fasciatus* and *T*.* vittatus*, also use shell patches for breeding [25]. *L*.* callipterus* is not aggressive toward *N*.* brevis* and tolerates their using the unoccupied shells for breeding. The shell patches are therefore communal nests. 

In our pilot study, mature males of different size classes were found in the shell patches, with larger males floating several dozen centimeters and sometimes more than 1 m above the patches and smaller males residing in the shells. This resembles the bourgeois “nesting” males and parasitic “dwarf” males of *L*.* callipterus* [18, 26, 36, 37]. Therefore, we suspected that the male* N*.* brevis* may also exhibit ARTs. If parasitic males are present in this fish population, it is expected that there exist discontinuous differences in aggressiveness and relative testes investment (testes mass relative to body mass) between males of the different size classes [8, 15, 18, 38]. In this study, we examined habitat use and the presence of ARTs in *N*.* brevis* in the Wonzye population.

## 2. Methods

### 2.1. Sampling

Field studies using SCUBA diving techniques were conducted at Wonzye Point (8°43′S, 31°08′E) near Mpulungu, Zambia, from November to December 2002, and October to November 2007. Where this population is found, the ground is covered with rocks from the shoreline to about 9 m depth (rocky habitat) or with sand to about 7–11 m depth (sandy bottom habitat). In this population, *N*.* brevis* occurred in both habitats, each of which was divided into two subhabitats: (1) sandy bottoms that were almost covered with shells at 9–11 m depth (662.5 shells/m^2^, [25]) (henceforth “shell bed” or SB); (2) sandy bottoms on which shells were widely distributed at 7–11 m depth (0.12 shells/m^2^, [25]) (henceforth “separated shells on sandy bottom” or SS); (3) midwater aggregations in rocky habitats that often consisted of >100 individuals, at 4–9 m depth (henceforth “midwater aggregation” or MA); (4) shell patches of *L*.* callipterus* in rocky habitats, which often consisted of >100 shells, at 4–9-m depth (shell density = 496.8 shells/m^2^, [25]) (henceforth “shell patch” or SP).

Habitat use was determined by differences in size structure, frequency of reproductively active individuals, and behavior among the habitats. To examine the size structure, we captured randomly selected *N*.* brevis* using gill nets in three habitats from November to December 2002 (*N*_SB_ = 60, *N*_SS_ = 63, *N*_MA_ = 34) and in one habitat from October to November 2007 (*N*_SP_ = 156, see below for details). We brought the collected fish back to the laboratory and measured their standard length (SL) to nearest 0.12 mm and body mass (BM) to nearest 0.002 g. All fish sampled from sandy bottom habitats and midwater aggregations and some of the fish sampled from the shell patches (*N*_male_ = 48, *N*_female_ = 31) were dissected immediately after sacrificing among crushed ice or by anesthetizing with eugenol. We then weighed their gonad mass (GM) to nearest 0.002 g and sexed individuals. Undissected females were released at the capture points. Male maturity was determined from developmental stages of gonads, because testes were either white and enlarged or transparent and threadlike. Males with white and enlarged testes were considered to be mature. Brood-caring females and females whose ovaries were filled with large, orange-colored (i.e., ripe) eggs were labeled as mature. Consequently, as the minimum size of mature females was 31.6 mm SL, we considered females ≥31.6 mm SL to be sexually mature, even if their ovaries were filled with unripe eggs. For the dissected fish (*N*_SB_ = 60, *N*_SS_ = 63, *N*_MA_ = 34, *N*_SP_ = 79), we calculated the gonad-free condition factor (i.e., BM-GM × 10^5^/SL^3^) and compared it among habitats and by sex. We also calculated the gonadosomatic index (GSI) of the dissected males as an estimate of testes investment and compared it among habitats (see below for details).

To examine the size structure and describe the reproductive ecology of *N*.* brevis*, we conducted an intensive field study in the shell patches in 2007. We determined the number of males and females in each shell patch, the maturity of males, and the breeding status of females from October to November 2007. We also examined whether there was a spawning cycle in* N*.* brevis, *which would be an important aspect of the reproductive ecology of this fish because *N*.* brevis* may have a periodic spawning cycle given that the reproductive cycle of the patch-owner species *L*.* callipterus* displays weak lunar-related periodicity [39]. Additionally, it was important to describe the mating system, as the different sampling days among habitats may have influenced reproductive parameters. In 17 randomly selected shell patches, we counted the numbers of *N*.* brevis *individuals that remained in shells in each patch once a week from 19 October to 12 November 2007, during which period a new and full moon occurred twice and once, respectively. Consequently, we obtained the counts for a continuous 6-week period in each shell patch. Because *N*.* brevis* resides in a shell in a head first position, the presence of an individual can be identified by observation of the caudal fin. We therefore visually checked whether *N*.* brevis* were present in shells by observing the entrance of shells. However, this visual counting could lead to an underestimation due to missing individuals that had retreated into the inner part of the shells or an overestimation due to the inclusion of males hiding in shells. To confirm the exact number of *N*.* brevis* and clarify the presence and absence of fish in a shell, all shells in the shell patches would need to be crushed. Because the application of this method is destructive, it was performed in only a portion of the shell patches (*N* = nine patches), minimizing the impact on the habitats. At the end of the observation (12 November 2007), we counted the number of shells in the nine shell patches by visual inspection and captured all shells and *N*.* brevis* individuals that were floating above the shell patches or that remained near the shells (*N* = 145). In the remaining eight nests, 11 floating *N*.* brevis* individuals were also captured. Hence, we sampled a total of 156 *N*.* brevis* in 2007. All fish and shells that were sampled were brought back to the laboratory. To determine the exact number of *N*.* brevis* individuals all collected shells were crushed using an iron hammer. The numbers revealed by visual counting and by actual counting (i.e., counting after crushing of shells) were strongly positively correlated (Poisson regression model, *χ* ^2^ = 52.42, df = 1, *P* < 0.001, *N* = 9), although visual counting had identified only three-quarters of the actual counting (mean ± SD = 74.0 ± 26.7%, *N* = 9). This indicates that the number of *N*.* brevis* estimated in the field by visual counting is a good index of the exact number of *N*.* brevis*, although with some underestimation.

### 2.2. Behavioral Observations

To examine the differences in behavioral characteristics among the four habitats, behavioral observations were conducted at a variety of locations in each habitat in November and December 2002. A total of 123 individuals that were randomly selected (*N*_SS_ = 22, *N*_SB_ = 19, *N*_MA_ = 23, *N*_SP_ = 59) were observed for 10 minutes, and the time spent in the shells (i.e., hiding), pecking behavior (i.e., feeding), aggressive behavior (i.e., dashing and fin spreading to opponents), and the opponents (conspecifics or heterospecifics) were recorded. Observed fish were later captured and sexed based on the shape of genital papilla and body color under water. They were released at the original capture points immediately after the sex determination.

### 2.3. Statistical Analyses

To examine the difference in size structure within and among habitats, we compared the SL of both sexes and the proportion of individuals available for mating (i.e., gravid females or mature males). For females, we compared the SL among three habitats (i.e., shell beds, separated shells on a sandy bottom, and shell patches) because only one female was sampled from midwater aggregations. For males, we compared the SL among all four habitats and between mature and immature individuals in each habitat. Comparisons were performed by general linear models (GLMs) fitted to a Gaussian distribution. The proportions of gravid females and mature males were compared between sandy and rocky habitats using Fisher's exact probability test. This analysis included fish samples that were taken in different years, which may have resulted in a difference of the size structure among habitats. However, because the periodic observations showed that temporal variation in reproductive activity was very little during the season (see results) and the environments within the littoral zone of Lake Tanganyika are considered to be stable over time [40], the differences generated by such effects were assumed to be negligible.

The periodicity of spawning was examined by the repeated-measures procedure, that is, a generalized linear mixed model fitted to a Poisson distribution (the number of females in shells = the dependent variable, date = the fixed factor, patch identifier = the random factor).

Other phenotypic data (the condition factor and testes investment) were also compared among habitats. The condition factor was compared for each sex, whereas the GSI was compared only for males using GLMs. In these comparisons, we took into account the differences between males in the shell patches, which may employ different reproductive tactics. For this purpose, we divided them into two groups based on adherence to shells, resulting in a total of five categories. To compare testes investment among habitats, we first attempted to follow the methodology of Tomkins and Simmons [17] in which log gonad mass (GM) was compared among groups, with log soma mass (BM-GM) as a covariate. In this analysis, only mature males were included (*N* = 128 males). There was a significant interaction between habitat and log soma mass (GLM, habitat × log soma mass: *F*_4,88_ = 3.570, *P* = 0.01). We therefore estimated testes investment by GSI.

 To examine differences in behavioral characteristics among habitats, behavior was also compared among three habitats for females and among four habitats for males. We also divided males in the shell patches into two groups based on adherence to shells as in the previous observations. Count data (the number of feeding events and attacks) were compared by use of a generalized linear model fitted to Poisson distribution models (Poisson GLMs). When the residual deviance divided by the degrees of freedom (deviance/df) of the model suggested overdispersion (deviance/df ≥ 2), we took account of this by fitting a generalized linear model to a negative binomial distribution (negative binomial GLM). We thereby succeeded in avoiding overdispersion (i.e., deviance/df < 2 for all models). Time spent in the shell was compared using the nonparametric Kruskal-Wallis test. 

For all multiple comparisons (i.e., comparisons among habitats), we corrected the significant values using the Bonferroni method so as to avoid type I errors. All analyses were performed using R 2.13.0.

## 3. Results

### 3.1. Size Structure

#### 3.1.1. Sandy-Bottom Habitats

**Figure 1:**
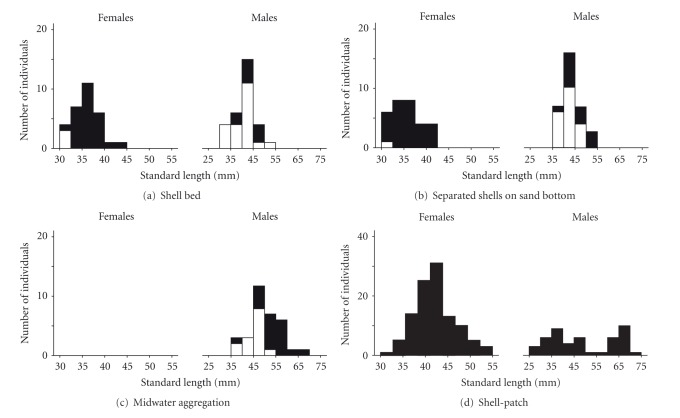
Body size histograms for *N*.* brevis *from four different habitats within the Wonzye population. Filled and blank bars indicate mature and immature individuals, respectively.

Sixty* N*. *brevis* were captured in the shell beds including 30 females and 30 males. We found only one gravid female (34.5 mm SL). Of the remaining females, three were immature and 26 were mature females that had ovaries filled with unripe eggs. Sexually matured males were 30% (*N* = 9) of all males captured in the shell beds. There was no difference in SL between mature (mean ± SD = 42.9 ± 3.9 mm) and immature males (40.1 ± 4.4 mm, GLM, *F*_1,28_ = 2.65, *P* = 0.12, Figure 1(a)). Males were larger than females (mean ± SD = 35.7 ± 2.8 mm SL, *N* = 30, GLM, *F*_1,58_ = 29.268, *P* < 0.001, Figure 1(a)).

Of 63 *N*.* brevis* captured in the separated shells on a sandy-bottom habitat, 30 were females and 33 were males. Gravid females were 47% (*N* = 14) of all females, and the other females captured either had ovaries filled with unripe eggs (*N* = 15) or were immature (*N* = 1). Sexually mature males constituted 42% (*N* = 14) of all males captured. Mature males (mean ± SD = 45.5 ± 4.3 mm SL) were larger than immature ones (41.8 ± 3.0 mm, GLM, *F*_1,31_ = 9.39, *P* = 0.007, Figure 1(b)). Males were larger than females (mean ± SD = 35.5 ± 3.2 mm SL, *N* = 30, GLM, *F*_1,61_ = 75.18, *P* < 0.001, Figure 1(b)). 

#### 3.1.2. Rocky Habitats

Midwater aggregations mainly consisted of males (33 of 34 fish captured). Sexually mature males accounted for 58% (*N* = 19) of all males captured. The one female captured was mature and had an ovary containing unripe eggs (SL = 36.4 mm). Mature males (mean ± SD = 52.6 ± 6.3 mm SL) were larger than immature ones (45.6 ± 4.2 mm, GLM, *F*_1,31_ = 12.95, *P* = 0.001, Figure 1(c)).

Visual counting indicated that a number of *N*.* brevis* individuals occupied shells within the shell patches throughout the 6-week observation period (mean ± SD = 9.5 ± 1.1 individuals/patch per week). There was no difference in the number of *N*.* brevis* individuals occupying shells over the 6-week observation period (Poisson mixed model, *χ* ^2^ = 10.87, df = 5, *P* > 0.05), indicating that there was no spawning cycle corresponding to the lunar cycle.

Actual counting (i.e., counting after the crushing of shells) determined that a mean number of 16.1 *N*. *brevis* (SD = 11.3) occurred in a shell patch, with both males and females present (females: mean ± SD = 12.0 ± 9.0, males: 4.1 ± 3.2, *N* = 9 patches). Of the females, half were brood-caring females (mean number ± SD = 5.9 ± 6.5, *N* = 9 patches) and the other half were gravid females that had not yet spawned (6.1 ± 4.4, *N* = 9 patches). Of the males, 40% were floating above the patches (mean number ± SD = 1.6 ± 0.7, *N* = 9 patches), whereas 60% remained close to or in the shells (2.6 ± 2.7, *N* = 9 patches). All of the males in both locations were sexually mature.

Floating males (mean ± SD = 60.4 ± 8.3 mm SL, *N* = 25) were much larger than males residing in shells (35.2 ± 4.6 mm SL, *N* = 23, GLM, *F*_1,46_ = 164.63, *P* < 0.001), which resulted in a bimodality in the size distribution of males found in the shell patches (Figure 1(d)). Females (40.9 ± 4.0 mm SL, *N* = 107) were larger than the males in shells but were smaller than floating males (GLM, *F*_2,152_ = 186.40, *P* < 0.001, after Bonferroni correction). Of the males in shells 78% (*N* = 23) were found solely in shells, and the others were found with brood-caring females in shells. In the later cases, males remained at the shells where females had spawned (*N* = 2) were spawning (*N* = 1) or had not yet spawned (*N* = 2). In all cases, males were always found in a head-first position nearer to the entrance than females. The former “solo” males (mean ± SD = 34.3 ± 4.5 mm SL) were marginally smaller than the latter “partnered” males (38.6 ± 3.8 mm SL, GLM, *F*_1,21_ = 3.65, *P* = 0.07).

#### 3.1.3. Comparisons among Habitats

Gravid females were more frequently found in rocky habitats (gravid : nongravid = 107 : 1) than in sandy-bottom habitats (gravid : nongravid = 15 : 45, Fisher's exact probability test, *P* < 0.001). Likewise, the proportion of mature males was greater in rocky habitats (mature : immature = 67 : 14) than in sandy bottom habitats (mature : immature = 23 : 40, Fisher's exact probability test, *P* < 0.001). Females in the shell patches were larger than those in sandy-bottom habitats (GLM, *F*_2,164_ = 40.01, *P*<0.001, after a Bonferroni correction, Figure 1). Male size likewise differed among habitats (GLM, *F*_4,138_ = 69.48, *P* < 0.001, males floating above the shell patches > male_MA_ > male_SB_ = male_SS_ > males in a shell within the shell patches, after a Bonferroni correction, Figure 1).

**Figure 2:**
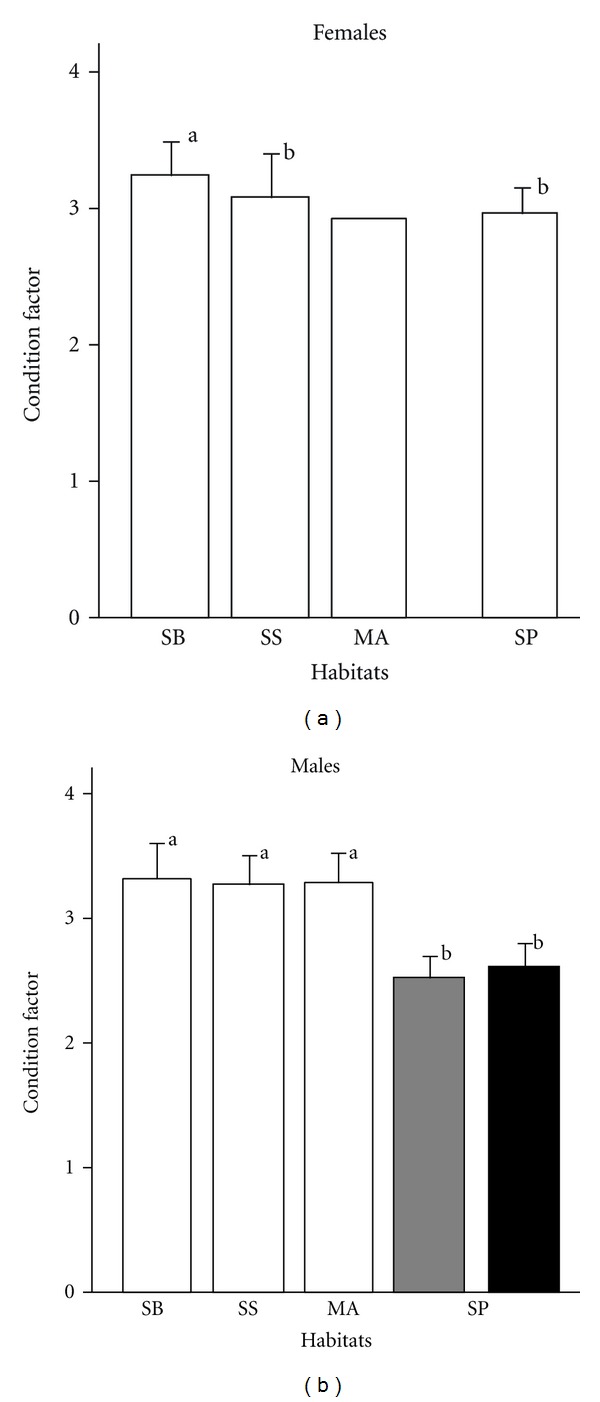
Comparisons of the condition factor among different habitats for each sex. SB, shell bed; SS, shells on a sandy bottom; MA, midwater aggregation in a rocky habitat; SP, shell patches in a rocky habitat. The single female in midwater aggregations was omitted from analysis. Filled and grey bars indicate males floating above nests and males in shells, respectively. Different letters beside the values indicate statistically significant differences as determined using a Bonferroni correction.

Females in the shell beds were in better condition than those in other habitats (GLM, *F*_1,88_ = 8.89, *P* < 0.001, after a Bonferroni correction, Figure 2). Likewise, there was a variation in the condition factor for males among habitats: all males in the shell patches were in poorer condition compared with males in other habitats (GLM, *F*_4,138_ = 75.06, *P* < 0.001, after a Bonferroni correction, Figure 2).

**Figure 3:**
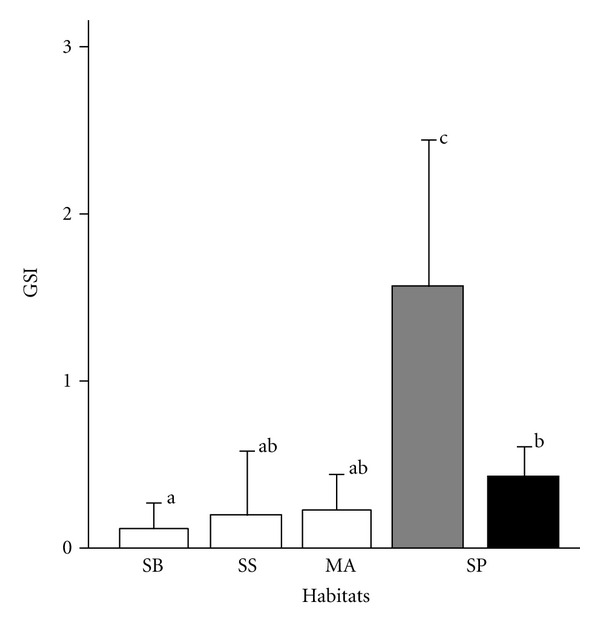
Comparisons of gonadosomatic index among the different habitats. SB: shell bed; SS, shells on a sandy bottom; MA: midwater aggregation in a rocky habitat; SP: shell patches in a rocky habitat. The single female in midwater aggregations was omitted from analysis. Filled and grey bars indicate males floating above nests and males in shells, respectively. Different letters beside the values indicate statistically significant differences determined using a Bonferroni correction.

There was also a great difference in testes investment (estimated by GSI) among habitats: males occupying shells within the shell patches had the greatest testes investment among males, and males in SB had the smallest testes investment (GLM, *F*_4,139_ = 49.49, *P* < 0.001, Figure 3).

### 3.2. Behavioral Observations

Females in the shell patches spent more time in shells than did those in sandy-bottom habitats (Kruskal-Wallis, test, *χ* ^2^ = 15.16, df = 2, *P* = 0.001, Table 1). Larger males in the shell patches seldom entered the shells, but this was not the case for the small males (Kruskal-Wallis, test, *χ* ^2^ = 82.58, df = 4, *P* < 0.001, Table 1). *N*.* brevis* individuals usually floated a few meters above the lake bottom in midwater aggregations, whereas in sandy-bottom habitats, individuals foraged in relative close proximity to (several dozen centimeters to a few meters above) the bottom where shells used for shelter were distributed. The feeding frequency of females was lower in the shell patches than in sandy-bottom habitats (negative binomial GLM, *χ* ^2^ = 9.78, df = 2, *P* < 0.01, Table 1). In accordance with this tendency of females, male feeding frequency was also lower in the shell patches than in the other habitats (negative binomial GLM, *χ* ^2^ = 66.51, df = 4, *P* < 0.001, Table 1).

Aggressive interactions between *N*.* brevis* females occurred only in the shell beds (Poisson GLM, *χ* ^2^ = 19.71, df = 2, *P* < 0.001, Table 1). Aggressive behavior toward other species was observed in sandy-bottom habitats but not in the shell patches (Poisson GLM, *χ* ^2^ = 16.59, df = 2, *P* < 0.001, Table 1). Male attacks on conspecifics were much more frequently performed by males floating above the patches and males in sandy-bottom habitats than by the small males in the shell patches and midwater aggregations (negative binomial GLM, *χ* ^2^ = 74.22, df = 4, *P* < 0.001, Table 1). A similar tendency was found in attacks on heterospecifics (Poisson GLM, *χ* ^2^ = 67.36, df = 4, *P* < 0.001, Table 1).

## 4. Discussion

**Figure 4:**
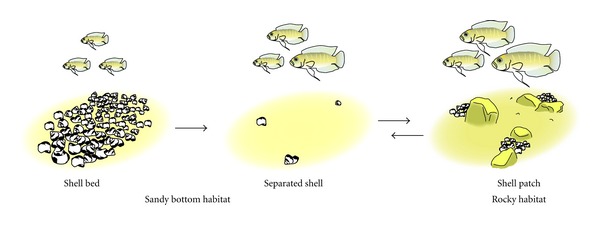
A schematic representation of the proposed scenario of life history in *N. brevis*. Arrows indicate the directions of fish movements.

*N*.* brevis* obligately uses empty gastropod shells for breeding and seldom uses substrates other than shells for shelter. Therefore, the distribution of shells is expected to strongly influence the distribution of *N*.* brevis*. *N*.* brevis* was found in every habitat of the Wonzye population where shells were present. However, breeding females occurred only in the shell patches. This indicates that the breeding events of* N*.* brevis* exclusively take place in the shell patches for this population. In contrast, mature males and gravid females were also found in sandy-bottom habitats, although much less frequently than in rocky habitats. Both sexes, with the exception of the small males in the shell patches, were smaller in sandy-bottom habitats than in rocky habitats. This suggests that *N*.* brevis* spend their early life-history stages in sandy-bottom habitats and migrate to shell patches for reproduction, possibly through shells distributed on the sand bottom between the shell bed and rocky habitats (Figure 4).

Such movements among habitats are often found in fish (e.g., [41, 42]) and are favored if the benefits exceed the costs [43–45]. In *N*.* brevis*, the shell patches may be a favorable habitat for breeding compared with other habitats. Shell patches contained a number of conspecific and heterospecific females in the Wonzye population (this study and [25]), and these patches likely enable more effective avoidance of egg predation through dilution effects [46] than would breeding in sandy-bottom habitats. However, the shell patches may be unsuitable for later growth and survival compared with other habitats. The complex structures of rocks, for example, would disturb food delivery (plankton drifting in the water column) to the shell patches. Consequently for feeding, *N*.* brevis* would be required to float in the midwater column, where the fish would be exposed to predation risk by piscivorous (e.g., *Lepidiolamprologus* spp. and* Lamprologus* spp.) and scale-eating (*Perissodus* spp.) fish. In contrast, sandy-bottom habitats may provide reduced predation risk and high food delivery near shelters. In these habitats, *N*.* brevis* can feed in close proximity to shells because there are no rocks disturbing food delivery, and individuals could hide in the shells immediately in response to the perceived risk of predation. Indeed, we found that *N*.* brevis* in the shell patches infrequently fed on plankton and consequently had poorer somatic condition compared with *N*.* brevis* individuals in other habitats. This suggests that sandy bottom habitats and midwater aggregations may be favored for* N*.* brevis* to invest in somatic growth. We therefore hypothesize that *N*.* brevis* chooses favorable habitats according to the stage of the life history. However, there is another possible explanation for the movements among habitats. In Wonzye, movements from shell-patches might be inevitable. *N*. *brevis* females who had finished broodcaring would be obstacle, because they would no longer yield any profits for *L*. *callipterus* (such as avoidance of egg predation through dilution effect, see above). If so, *L*.* callipterus* would expel them so as to secure shells for their own breeding. Then, *N*.* brevis* individuals would be forced to migrate to near other habitats, that is, separated shells on sandy bottom. These hypotheses are not mutually exclusive, and it is therefore possible that both factors are responsible for movements among habitats.

Several males were found within the shell patches at the same time. These males were divided into two types based on morphological and behavioral phenotypes that were clearly differentiated. Males floating above the shell patches were larger in body size and exhibited relatively frequent aggressive behavior toward conspecifics. Males who usually stayed close to or in the shells were smaller and less aggressive. These discontinuous differences in phenotypes are found in the related shell-brooding cichlids with ARTs, *L*.* callipterus* [3, 26, 37], and *T*.* vittatus* [30]. In *L*.* callipterus*, there are two types of reproductively parasitic males: “sneaker” males dart toward the shell where spawning takes place and ejaculate from the entrance of the shell and “dwarf” males wriggle past spawning females and take up residence behind them so as to ejaculate in close proximity to females [37]. In *T*.* vittatus*, sneaker males get inside the shell for several seconds or longer and ejaculate, and they then leave the shell immediately [30]. In* N*.* brevis*, most sexually mature small males were found solely in shells where spawning had not yet taken place. Although these males may be simply hiding in the shells, it is possible that they may have a sit-and-wait tactic for “sneaking” in which they lie in wait for a female to spawn [37]. Because the sit-and-wait tactic enables *N*.* brevis* males to position themselves behind females, smaller bodies are required to succeed with this approach. Indeed, these males were marginally smaller than the males that remained in shells with females. We also found several males entering the shells after females, some of which had not yet mated. Additionally, small male* N*.* brevis* individuals were found behind females in shells (i.e., in the innermost parts of the shells, [34]). This indicates the presence of wriggling “sneaking” by small male* N*.* brevis*. Together, these observations indicate that small mature male *N*.* brevis* may have a wide range of parasitic “sneaky” behavior types so as to steal fertilization opportunities.

If the small males really do employ parasitic tactics, their ejaculation should usually occur during mating by territorial males. In this situation, theory predicts that their testes investment is greater than territorial conspecifics [8]. This was the case in *N*.* brevis* as well as other Lake Tanganyika cichlids employing ARTs [18, 21, 22, 26, 28, 30, 37], but some controversy about this remains (see [47]). Testes investment among the floating males was the same as for males found in the midwater and sandy-bottom habitats and was much smaller than that of males within the shells of the shell patches. These findings are interpreted as demonstrating the presence of ARTs, although we did not directly observe the spawning behavior of *N*.* brevis*.

Several territorial males were found within some of the shell patches. Together with the finding of a number of females in the shell patches, this suggests that *N*. *brevis *exhibits multimale polygyny [48], in accordance with a previous study [25]. This mating system differed from that of other shell brooders found in the shell patches: both *L*.* callipterus* [37] and* T*.* vittatus* [30] are polygynous. The hierarchy among these species and the reason they share a shell patch remain to be investigated. Further studies that include paternity analysis are needed to clarify the mating system of *N*.* brevis.*

In conclusion, the results strongly suggest that the mating system of *N*.* brevis* is characterized by movements among habitats and multimale polygyny with ARTs. ARTs seem to be a common attribute in the Lake Tanganyika cichlid tribe Lamprologini, particularly in shell brooders. It is possible that the specific shape of the empty gastropod shells is at least partly responsible for this universality. Several studies have suggested that the presence of a space in a breeding substrate that large bourgeois males cannot enter is an important factor allowing “sneakers” to gain access to females [23, 28, 37]. As the entrance of the shells is very small (3.3 cm^2^ on average in Wonzye), entering the shells requires specialized morphologies (e.g., small body size [27, 37]). However, territorial males are selected for large size because of the intense male-male competition and, particularly in case of *L*.* callipterus*, the requirements of shell transportation (e.g., [49–51]). This difference in optimal body size between tactics leads to biased access to the shells: “sneakers” can penetrate the spawning substrate to a depth that territorial males cannot reach. In accordance with this pattern, in *N*.* brevis*, smaller males entered shells, whereas large territorial males seldom entered. We therefore assume that the shape of a breeding substrate is one of the key factors leading to the prevalence of ARTs among the Lake Tanganyika shell-brooding cichlids.

## Figures and Tables

**Table 1:** Differences in four behavior types for male *N*.  *brevis* within and among habitats.

	Habitats				
	Sand-bottom habitats		Rocky habitats		
	Shell bed	Separated shell	Aggregation	Shell patch	
Females	(*N* = 7)	(*N* = 3)		(*N* = 14)	
Time spent in shell (min)	0.28 ± 0.50^a^	3.33 ± 5.77^ab^		8.34 ± 2.82^b^	
Number of feeding pecks	76.9 ± 35.6^a^	73.3 ± 69.4^a^		0.36 ± 1.33^b^	
Number of intraspecific attacks	1.14 ± 1.57^a^	0^b^		0^b^	
Number of heterospecific attacks	0.71 ± 0.76^a^	1.33 ± 1.53^ab^		0^b^	

Males	(*N* = 12)	(*N* = 19)	(*N* = 23)	(*N* = 22)^†^	(*N* = 23)^‡^
Time spent in shell (min)	0.09 ± 0.21^a^	0^a^	0^a^	0.01 ± 0.04^a^	4.78 ± 3.55^b^
Number of feeding pecks	48.5 ± 25.6^ab^	82.8 ± 100.1^a^	57.2 ± 40.5^a^	11.5 ± 24.7^b^	1.3 ± 3.1^c^
Number of intraspecific attacks	3.3 ± 4.5^a^	2.5 ± 2.0^a^	0^b^	3.8 ± 3.9^a^	0.09 ± 0.29^b^
Number of heterospecific attacks	1.17 ± 1.64^a^	1.58 ± 3.08^a^	0.04 ± 0.21^b^	0.77 ± 0.87^a^	0.04 ± 0.21^b^

Values are means ± SD.

Different letters beside the value indicate statistical significances determined using the Bonferroni correction.

^†^Males floating above shell patches.

^‡^Males found in shell of shell patches.

## References

[1] Taborsky M (1994). Sneakers, satellites, and helpers: parasitic and cooperative behavior in fish reproduction. *Advances in the Study of Behavior*.

[2] Taborsky M (1997). Bourgeois and parasitic tactics: do we need collective, functional terms for alternative reproductive behaviours?. *Behavioral Ecology and Sociobiology*.

[3] Taborsky M (2001). The evolution of bourgeois, parasitic, and cooperative reproductive behaviors in fishes. *Journal of Heredity*.

[4] Gross MR (1996). Alternative reproductive strategies and tactics: diversity within sexes. *Trends in Ecology and Evolution*.

[5] Shuster SM, Wade MJ (2003). *Mating Systems and Strategies*.

[6] Oliveira RF, Taborsky M, Brockmann HJ (2008). *Alternative Reproductive Tactics: An Integrative Approach*.

[7] Parker GA, Birkhead TR, Møller AP (1998). Sperm competition and the evolution of ejaculates: towards a theory base. *Sperm Competition and Sexual Selection*.

[8] Parker GA (1990). Sperm competition games: sneaks and extra-pair copulations. *Proceedings of the Royal Society B*.

[9] Gage MJ, Stockley P, Parker GA (1995). Effects of alternative male mating strategies on characteristics of sperm production in the Atlantic salmon (*Salmo salar*): theoretical and empirical investigations. *Philosophical Transactions of the Royal Society B*.

[10] Simmons LW, Tomkins JL, Hunt J (1999). Sperm competition games played by dimorphic male beetles. *Proceedings of the Royal Society B*.

[11] Leach B, Montgomerie R (2000). Sperm characteristics associated with different male reproductive tactics in bluegills (*Lepomis macrochirus*). *Behavioral Ecology and Sociobiology*.

[12] Uglem I, Rosenqvist G, Wasslavik HS (2000). Phenotypic variation between dimorphic males in corkwing wrasse. *Journal of Fish Biology*.

[13] Vladić TV, Järvi T (2001). Sperm quality in the alternative reproductive tactics of Atlantic salmon: the importance of the loaded raffle mechanism. *Proceedings of the Royal Society B*.

[14] Neff BD, Fu P, Gross MR (2003). Sperm investment and alternative mating tactics in bluegill sunfish (*Lepomis macrochirus*). *Behavioral Ecology*.

[15] Drilling CC, Grober MS (2005). An initial description of alternative male reproductive phenotypes in the bluebanded goby, *Lythrypnus dalli* (Teleostei, Gobiidae). *Environmental Biology of Fishes*.

[16] Taborsky M, Oliveira RF, Taborsky M, Brockmann HJ (2008). Alternative reproductive tactics in fish. *Alternative Reproductive Tactics*.

[17] Tomkins JL, Simmons LW (2002). Measuring relative investment: a case study of testes investment in species with alternative male reproductive tactics. *Animal Behaviour*.

[18] Ota K, Kohda M, Hori M, Sato T (2011). Parker’s sneak-guard model revisited: why do reproductively parasitic males heavily invest in testes?. *Naturwissenschaften*.

[19] Sefc KM (2011). Mating and parental care in lake tanganyika's cichlids. *International Journal of Evolutionary Biology*.

[20] Haesler MP, Lindeyer CM, Taborsky M (2009). Reproductive parasitism: male and female responses to conspecific and heterospecific intrusions at spawning in a mouth-brooding cichlid Ophthalmotilapia ventralis. *Journal of Fish Biology*.

[21] Awata S, Heg D, Munehara H, Kohda M (2006). Testis size depends on social status and the presence of male helpers in the cooperatively breeding cichlid Julidochromis ornatus. *Behavioral Ecology*.

[22] Awata S, Takeyama T, Makino Y, Kitamura Y, Kohda M (2008). Cooperatively breeding cichlid fish adjust their testis size but not sperm traits in relation to sperm competition risk. *Behavioral Ecology and Sociobiology*.

[23] Kohda M, Heg D, Makino Y (2009). Living on the wedge: female control of paternity in a cooperatively polyandrous cichlid. *Proceedings of the Royal Society B*.

[24] Dierkes P, Taborsky M, Achmann R (2008). Multiple paternity in the cooperatively breeding fish *Neolamprologus pulcher*. *Behavioral Ecology and Sociobiology*.

[25] Gashagaza TSatoandMM, Kawanabe H, Hori M, Nagoshi M (1997). Shell-brooding cichlid fishes of Lake Tanganyika: their habitats and mating systems. *Fish communities in Lake Tanganyika*.

[26] Schütz D, Pachler G, Ripmeester E, Goffinet O, Taborsky M (2010). Reproductive investment of giants and dwarfs: specialized tactics in a cichlid fish with alternative male morphs. *Functional Ecology*.

[27] Ota K, Kohda M, Sato T (2010). Why are reproductively parasitic fish males so small?-influence of tactic-specific selection. *Naturwissenschaften*.

[28] Katoh R, Munehara H, Kohda M (2005). Alternative male mating tactics of the substrate brooding cichlid *Telmatochromis temporalis* in Lake Tanganyika. *Zoological Science*.

[29] Mboko SK, Kohda M (1999). Piracy mating by large males in a monogamous substrate-breeding cichlid in Lake Tanganyika. *Journal of Ethology*.

[30] Ota K, Kohda M (2006). Description of alternative male reproductive tactics in a shell-brooding cichlid, *Telmatochromis vittatus*, in Lake Tanganyika. *Journal of Ethology*.

[31] Ota K, Hori M, Kohda M (2012). Changes in reproductive life-history strategies in response to nest density in a shell-brooding cichlid, *Telmatochromis vittatus*. *Naturwissenschaften*.

[32] Sunobe T, Munehara H (2003). Mating system and kin relationship between adults and young in the shell-brooding cichlid fish *Neolamprologus meeli* in Lake Tanganyika. *Journal of Ethology*.

[33] Sefc KM, Mattersdorfer K, Sturmbauer C, Koblmüller S (2008). High frequency of multiple paternity in broods of a socially monogamous cichlid fish with biparental nest defence. *Molecular Ecology*.

[34] Aibara M (2005). *Biology and taxonomy of Neolamprologus brevis species complex from LakeTanganyika (Perciformes: Cichlidae) [Ph.D. thesis]*.

[35] Konings A (1998). *Tanganyika Cichlids in Their Natural Habitats*.

[36] Sato T (1994). Active accumulation of spawning substrate: a determinant of extreme polygyny in a shell-brooding cichlid fish. *Animal Behaviour*.

[37] Sato T, Hirose M, Taborsky M, Kimura S (2004). Size-dependent male alternative reproductive tactics in the shell-brooding cichlid fish *Lamprologus callipterus* in Lake Tanganyika. *Ethology*.

[38] Gross MR (1982). Sneakers, satellites and parentals: polymorphic mating strategies in North American sunfishes. *Zeitschrift fur Tierpsychologie*.

[39] Nakai K, Yanagisawa Y, Sato T, Niimura Y, Gashagaza MM (1990). Lunar synchronization of spawning in cichlid fishes of the tribe Lamprologini in Lake Tanganyika. *Journal of Fish Biology*.

[40] Takeuchi Y, Ochi H, Kohda M, Sinyinza D, Hori M (2010). A 20-year census of a rocky littoral fish community in Lake Tanganyika. *Ecology of Freshwater Fish*.

[41] Hidaka T, Takahashi S (1987). Reproductive strategy and interspecific competition in the Lake-living gobiid fish isaza, *Chaenogobius isaza*. *Journal of Ethology*.

[42] Werner EE, Hall DJ (1988). Ontogenetic habitat shifts in bluegill: the foraging rate-predation risk trade-off. *Ecology*.

[43] Gross MR (1987). Evolution of diadromy in fishes. *American Fisheries Society Symposium*.

[44] McDowall RM (1997). The evolution of diadromy in fishes (revisited) and its place in phylogenetic analysis. *Reviews in Fish Biology and Fisheries*.

[45] Pittman SJ, McAlpine CA (2003). Movements of marine fish and decapod crustaceans: process, theory and application. *Advances in Marine Biology*.

[46] Johnston CE (1994). Nest association in fishes: evidence for mutualism. *Behavioral Ecology and Sociobiology*.

[47] Fitzpatrick JL, Desjardins JK, Stiver KA, Montgomerie R, Balshine S (2006). Male reproductive suppression in the cooperatively breeding fish *Neolamprologus pulcher*. *Behavioral Ecology*.

[48] Clutton-Brock TH (1989). Mammalian mating systems. *Proceedings of the Royal Society of London, Series B*.

[49] Schütz D, Parker GA, Taborsky M, Sato T (2006). An optimality approach to male and female body sizes in an extremely size-dimorphic cichlid fish. *Evolutionary Ecology Research*.

[50] Schütz D, Taborsky M (2005). The influence of sexual selection and ecological constraints on an extreme sexual size dimorphism in a cichlid. *Animal Behaviour*.

[51] Ota K, Kohda M, Sato T (2010). Unusual allometry for sexual size dimorphism in a cichlid where males are extremely larger than females. *Journal of Biosciences*.

